# Antimicrobial properties of lactic acid bacteria isolated from traditional yogurt and milk against Shigella strains

**DOI:** 10.3205/dgkh000307

**Published:** 2018-01-16

**Authors:** Elnaze Zare Mirzaei, Elahe Lashani, Abolfazl Davoodabadi

**Affiliations:** 1 Student Research Committee, Babol University of Medical Science, Babol, Iran; 2 Department of Microbiology, Medical School, Babol University of Medical Science, Babol, Iran; 3 Department of Microbiology, Medical School, Tehran University of Medical Science, Tehran, Iran

**Keywords:** lactic acid bacteria, yogurt, milk, antimicrobial properties, Shigella

## Abstract

**Background:** Lactic acid bacteria (LAB) are normal flora of the mouth, intestines and the female genital tract. They are also frequently found in meat, vegetables, and dairy products. Most of probiotic bacteria belong to the LAB group. Some probiotic LAB are useful in prevention and treatment of diarrheal diseases. The aim of this study was to investigate the antimicrobial properties of LAB isolated from traditional yogurt and milk against *Shigella* strains.

**Materials and methods:** Forty LAB strains were isolated from traditional yogurt and milk. The antimicrobial activity of LAB against *Shigella* strains (eight *S. flexneri*, four *S. sonnei*) was examined using the agar-well diffusion assay. LAB strains with antimicrobial effect against all *Shigella* strains were identified by 16S rRNA gene sequencing.

**Results:** Six LAB strains inhibited the growth of all 12 *Shigella* strains. *Lb. paracasei* Y1-3, *Lb. paracasei* Y8-1 and *Lb. fermentum* Y2-2 were isolated from yogurt. *Lb. paracasei* M18-1, *Lb. parelimentarius* M4-3 and *Lb. plantarum* M19-1 were isolated from milk.

**Conclusion:** This study showed that *Lactobacillus* strains with good inhibitory activity against *S. flexneri* and *S. sonnei* could be isolated from traditional yogurt and milk.

## Zusammenfassung

**Hintergrund:** Milchsäurebakterien (LAB) gehören zur normalen Flora der Mundhöhle, des Darmtrakts und des weiblichen Genitaltrakts. Sie sind häufig in Fleisch, Gemüse und Milchprodukten nachweisbar. Die meisten probiotischen Bakterien gehören zu den LAB. Einige probiotische LAB sind zur Prävention und Behandlung infektiöser Diarrhoen geeignet. Zielsetzung der Studie war die Untersuchung antimikrobieller Eigenschaften von aus traditionellem Joghurt und Milch isolierten LAB gegen Shigella-Stämme.

**Materialien und Methode:** 40 LAB-Stämme wurden aus traditionellem Joghurt und Milch isoliert. Die antimikrobielle Wirkung der LAB gegen die Shigella-Stämme (acht *S. flexneri*, vier *S. sonnei*) wurde im Agardiffusionstest ermittelt. Antimikrobiell wirksame LAB Stämme wurden mittels 16S rRNA Gensequenzing identifiziert.

**Ergebnisse:** Sechs LAB Stämme hemmten die Vermehrung der geprüften 12 Shigella Stämme. *Lactobacillus* (Lb.) *paracasei *Y1-3, *Lb. paracasei *Y8-1 und *Lb. fermentum *Y2-2 wurden aus Joghurt, *Lb. paracasei *M18-1, *Lb. parelimentarius *M4-3 und *Lb. plantarum *M19-1 aus Milch isoliert.

**Schlussfolgerung:** Aus Joghurt und Milch konnten Lactobacillus-Stämme mit guter Hemmwirkung gegen *S. flexneri* und *S. sonnei* isoliert werden.

## Background

Lactic acid bacteria (LAB) are normal, physiological flora of the mouth, intestines and female genital tract. They are also frequently found in meat, vegetables, and dairy products, such as milk and yogurt. The* Lactobacillus* genus is one of the most important genera in the group of LAB. *Lactobacillus* strains are Gram-positive, catalase-negative, non-spore-forming and non-motile bacilli [1], [2], [3]. LAB have protective effects in fermented food preservation, because they produce organic acids in food during their growth. Conversion of carbohydrates to organic acids and reduction of pH is the reason for the increased half-life and good quality of such food products [1], [4]. 

Probiotics are living microorganisms which, when consumed in adequate amounts, confer health benefits to the host by altering the indigenous microflora [5]. Most probiotic bacteria belong to the LAB group [6]. The presence of LAB in food has beneficial effects on human health, including effects on the natural gut microflora equilibrium, reducing blood cholesterol, decreasing intestinal tumors, facilitating calcium absorption in the intestines, reducing lactose intolerance, and preventing and treating diarrheal diseases [7], [8], [9]. Several mechanisms have been suggested for the inhibitory activity of LAB against pathogenic bacteria, especially Gram-negative pathogens. These mechanisms include production of organic acids, hydrogen peroxide and bacteriocin, and competition for colonization sites with pathogenic bacteria [10], [11], [12].

*Shigella* spp. are common intestinal Gram-negative pathogens which cause diarrheal diseases and dysentery in many countries [13]. *Shigella* spp. are a leading cause of gastroenteritis-induced deaths in 3–5 million children under five years old in developing countries [14]. In several studies, the probiotic potential of various *Lactobacillus* strains has been demonstrated. Some *Lactobacillus* strains commonly used as probiotics are efficaceous especially against acute diarrhea in children [11], [15], [16], [17]. The purpose of this study was to investigate the antimicrobial effect of LAB isolated from traditional yogurt and milk against *Shigella* spp.

## Methods

### Samples collection and culture

Twenty samples of traditional yogurt and milk were collected in sterile containers and transferred to the microbiology laboratory of Babol University of Medical Sciences, Iran. Two grams of yogurt and 500 µl of milk were inoculated into 15 ml de Man, Rogosa and Sharp (MRS) broth medium (Merck, Germany), and cultured for 48 h in anaerobic jars at 37°C. Then the MRS broth was subcultured on MRS agar (Merck, Germany) plates, and inoculated for 48 h in anaerobic jars at 37°C. The suspicious colonies were tested using Gram staining and catalase reaction. Gram-positive and catalase-negative bacteria were purified by streaking on MRS agar, and stocked in MRS broth containing 20% glycerol at –20°C.

### Evaluation of antimicrobial effect of LAB against Shigella strains

The antimicrobial activity of LAB against *Shigella* strains was tested using the agar well diffusion assay [18]. Twelve *Shigella* strains (eight *S. flexneri*, four *S. sonnei*), which were previously isolated from children with diarrhea, were included in this study. The LAB were cultured in 3 ml MRS broth medium in anaerobic jars and incubated for 24 h at 37°C. The MRS broth tubes were subsequently centrifuged (10,000 g, 10 min) to prepare cell-free culture supernatants (CFCS). *Shigella* strains were grown on nutrient agar medium (Merck, Germany) and incubated for 24 h at 37°C. A suspension of 10^7^ colony forming units (CFU)/ml of *Shigella* strains was then prepared and spread onto the nutrient agar, into which 5-mm-deep wells had been dug. About 100 µl of CFCS was poured into each well, and nutrient agar plates were incubated for 18 h at 37°C. *Lb. rhamnosus* GG was used as the positive control. Finally, inhibition zone diameter was measured. LAB strains with clear zones <11 mm, 11–16 mm, 17–22 mm and >23 mm, were classified as negative (–), mild (+), strong (++), and very strong (+++) inhibitors, respectively. 

### Production of bacteriocin-like compounds

Two main mechanisms of antimicrobial activity are the production of organic acids, which reduce the pH, and the production of hydrogen peroxide. Some *Lactobacillus* spp. also produce bacteriocin, which is antimicrobially active [19]. For these reasons, the pH of the CFCS was measured and adjusted to 6.5 with NaOH (Merck, Germany, 2.5M); catalase (1 mg/ml, Sigma-Aldrich, Germany) was then added to the CFCS and incubated at 25°C for 1 h [12]. The antimicrobial activity of these CFCS was investigated using the agar well diffusion assay.

### Identification of LAB species by 16S rRNA gene sequencing

LAB strains with antimicrobial efficacy against all *Shigella* strains were identified by 16S rRNA gene sequencing. DNA extraction of strains was performed using the boiling method [20]. PCR was performed with primers 27F (5'-CTCGTTGCGGGACTTAA-3') and 1522R (5'-GCAGCAGTAGGGAATCTTC-3') (Bioneer, Korea) [21]. The reaction mixture consisted of 1.5 mM MgCl_2_, 0.2 mM dNTPs, 2.75 ml of genomic DNA, 5 ml 10X PCR buffer, 3 pmol of each primer and 3 U of Taq DNA polymerase (Jena Bioscience, Germany) in a final volume of 50 ml. The PCR protocol started with an initial denaturation at 95°C for 2 min, followed by 35 cycles of 94°C for 1 min, 55°C for 1 min and 72°C for 10 min. PCR products were electrophoretically separated on agarose gel (1.5% w/v) and visualized by staining with safe stain (Yekta Tajhiz Azma, Iran). Finally, PCR products were sent for sequencing (Bioneer, Korea). Species of LAB were identified after comparison of the obtained sequences by nucleotide-nucleotide BLAST (https://www.ncbi.nlm.nih.gov/blast).

## Results

**Figure 1:**
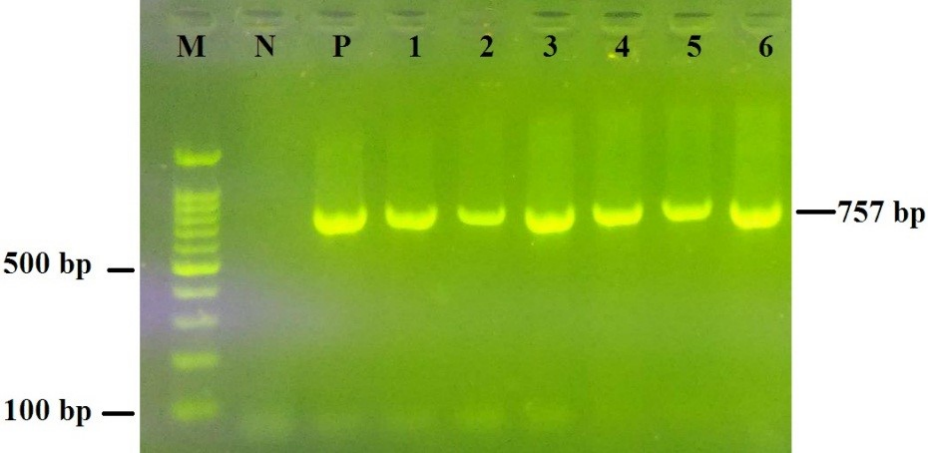
16s rRNA gene PCR. Lane M, 100 bp DNA ladder (Yekta Tajhiz Azma, Iran); Lane N, Negative control; Lane P, Positive control (*Lb. rhamnosus* GG); Lanes 1–6, *Lactobacillus* strains.

Forty LAB strains were isolated from 20 samples of traditional yogurt and milk (10 samples of milk and 10 samples of yogurt). Of the 40 LAB strains, 22 were obtained from milk and 18 strains from yogurt. Six LAB strains in the well diffusion assay inhibited the growth of all 12 *Shigella* strains (Table 1 (Tab. 1)). The species of these six LAB strains were identified by 16s rRNA gene sequencing PCR (Figure 1 (Fig. 1)). Three strains belonged to the species *Lb. paracasei*. *Lb. paracasei* Y1-3 (GenBank accession number KY552923.1) and *Lb. paracasei* Y8-1 (GenBank accession number: KY552924.1) were isolated from yogurt, and *Lb. paracasei* M18-1 (GenBank accession number: KY552927.1) was obtained from milk. Strain M4-3, which was isolated from milk, was identified as *Lb. parelimentarius* (GenBank accession number: KY552927.1). *Lb. fermentum* Y2-2 (GenBank accession number: KY552925.1) was isolated from yogurt. *Lb. plantarum* M19-1 (GenBank accession number: KY552926.1) was isolated from milk. 

*Lb. rhamnosus* GG has mild inhibitory activity against *Shigella* strains, while *Lb. paracasei* M18-1, *Lb. paracasei* Y8-1 and *Lb. plantarum* M19-1 have strong or very strong inhibitory activity against *Shigella* strains (Table 1 (Tab. 1)).

Among the other 34 strains of lactic acid bacteria, 6 inhibited the growth of 11 *Shigella* strains (91.6%), 9 inhibited the growth of 10 *Shigella* strains (83.3%), 5 inhibited the growth of nine *Shigella* strains (75%), 3 inhibited the growth of eight *Shigella* strains (66.6%), and 2 inhibited the growth of seven *Shigella* strains (58.3%) (data not shown).

The antimicrobial activity of *Lactobacillus* strains disappeared when the CFCS was adjusted to pH 6.5 and treated with catalase. The *Lactobacillus* strains did not produce bacteriocin-like compounds.

## Discussion

Shigellosis is usually a self-limiting infection, but in severe cases of the infection, antibiotic therapy may be required. Quinolones and cephalosporins are the drugs of choice. However, the worldwide emergence of antimicrobial resistance among *Shigella* species has limited the choice of antimicrobial agents for treating the infection. Resistance to different antibiotics such as sulfonamides, ampicillin, tetracyclines and co-trimoxazole has been described among *Shigella* species worldwide, and the causes of treatment failure can be attributed to these antibiotic resistances [13]. Because there are concerns about the increase in drug-resistant pathogenic bacteria, probiotic LAB are being used as a preventive treatment alternative [22]. Probiotic bacteria have been widely studied over the past decades in the prevention and treatment of diarrheal diseases. The most commonly used probiotic microorganisms for prevention and treatment of diarrheal diseases are *Lactobacillus* GG, *Lb. acidophilus*, *Lb. casei*, *Bifidobacterium* spp., *Streptococcus* spp., and the yeast *Saccharomyces boulardii* [23].

In this study, six *Lactobacillus* strains (three *Lb. paracasei*, one *Lb. parelimentarius*, one *Lb. fermentum*, and one *Lb. plantarum*) with inhibitory activity against all *S. flexneri* and *S. sonnei* strains were isolated from traditional yogurt and milk. Some of these *Lactobacillus* strains, such as *Lb. paracasei* M18-1, *Lb. paracasei* Y8-1 and *Lb. plantarum* M19-1 have strong or very strong inhibitory activity against *Shigella* strains. 

Mirnejad et al. [14] reported that CFCS from *Lb. casei* strongly inhibits the growth of multiple drug resistance (MDR) clinical samples of *S. sonnei* and *S. flexneri* in vitro. They suggested that *Lb. casei* is a good probiotic candidate. Zhang et al. [10] isolated 91 lactobacilli from human fecal samples and screened these lactobacilli for inhibitory activity against *S. sonnei*. Their results showed that 15 lactobacilli have strong inhibitory activity. Similar to our study, Hütt et al. [24] showed that *Lb. rhamnosus* GG has inhibitory activity against *S. sonnei* ATCC 25931. In addition, Zhihui Yu et al. [25] isolated *Lb. plantarum* S4-1 from naturally-fermented Chinese sauerkraut, and showed that this strain possesses inhibitory activity against *S. flexneri* CMCC. They suggested that *Lb. plantarum* S4-1 have potential as an excellent probiotic candidate for use in functional products.

In the present study, when the CFCS of *Lactobacillus* was adjusted to pH 6.5 and treated with catalase, the antimicrobial activity disappeared. These results suggest that the production of bacteriocin-like compounds did not play a role in the mechanism of antimicrobial activity. The inhibition of *Shigella* strains appeared to be the result of organic acids or hydrogen peroxide production by the *Lactobacillus* strains. Several studies have previously shown that a pH-dependent mechanism was involved in the antimicrobial activity of *Lactobacillus* strains [26], [27].

## Conclusions

*Lactobacillus* strains with good inhibitory activity against *S. flexneri* and *S. sonnei* were isolated from traditional Iranian yogurt and milk. These *Lactobacillus* strains may be useful as potential novel and effective probiotic strains for the prevention or treatment of diarrhea, but further in vitro and in vivo investigations on these strains are still required.

## Notes

### Competing interests

The authors declare that they have no competing interests.

### Acknowledgment

This work was supported by the Student Research Committee of Babol University of Medical Science, Babol, Iran (Grant no. 3976).

## Erratum

The first name of the author Davoodabadi was originally mispelled (Aboldfazl).

## Figures and Tables

**Table 1:**
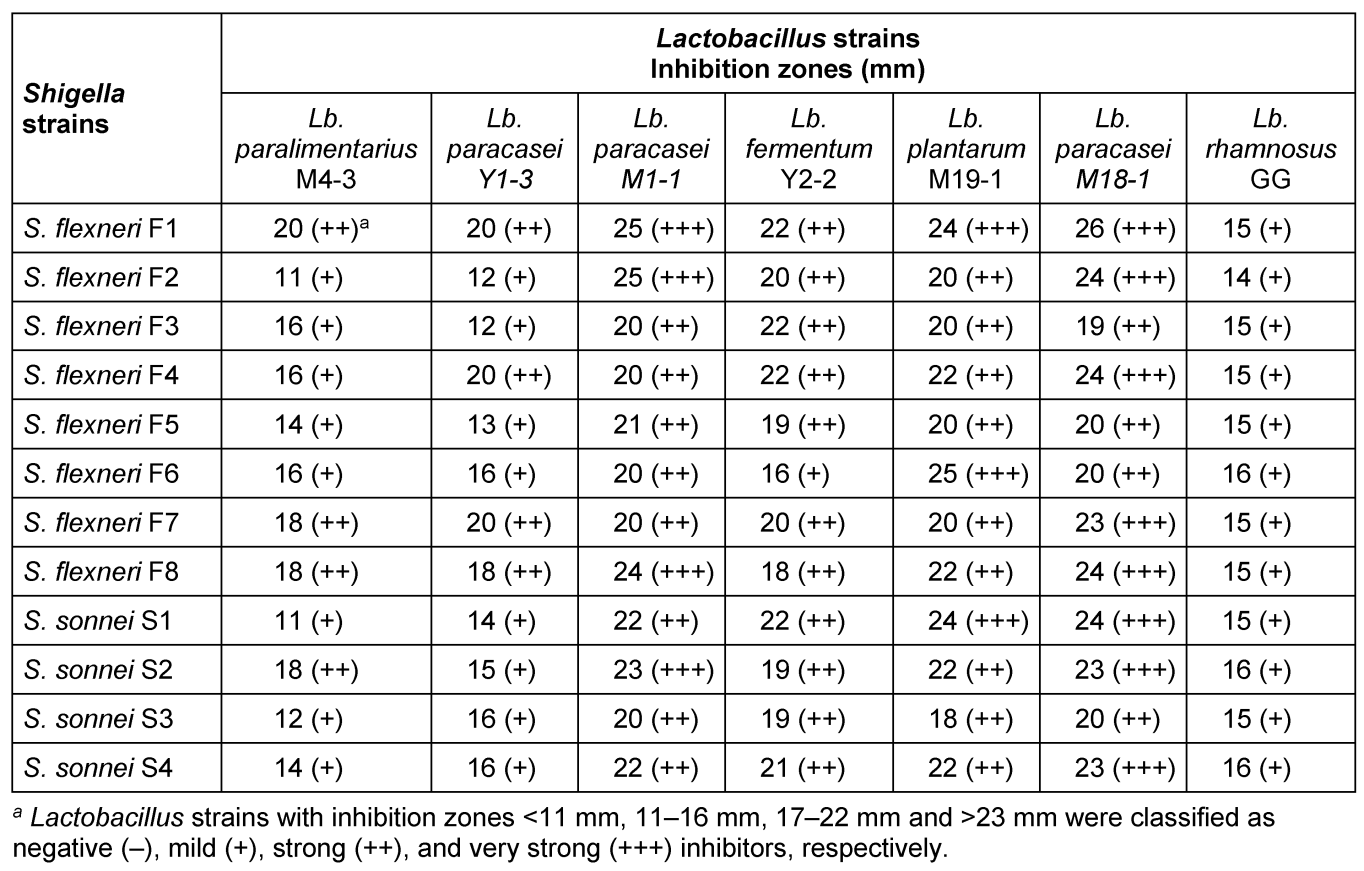
Inhibitory activity of cell-free culture supernatants (CFCS) of *Lactobacillus* strains against *Shigella* strains
